# Refining trophic dynamics through multi‐factor Bayesian mixing models: A case study of subterranean beetles

**DOI:** 10.1002/ece3.6580

**Published:** 2020-07-20

**Authors:** Mattia Saccò, Alison J. Blyth, William F. Humphreys, Steven J. B. Cooper, Andrew D. Austin, Josephine Hyde, Debashish Mazumder, Quan Hua, Nicole E. White, Kliti Grice

**Affiliations:** ^1^ WA‐Organic Isotope Geochemistry Centre The Institute for Geoscience Research School of Earth and Planetary Sciences Curtin University Perth WA Australia; ^2^ Collections and Research Centre Western Australian Museum Welshpool WA Australia; ^3^ School of Biological Sciences University of Western Australia Crawley WA Australia; ^4^ Australian Centre for Evolutionary Biology and Biodiversity School of Biological Sciences University of Adelaide Adelaide SA Australia; ^5^ Evolutionary Biology Unit South Australian Museum Adelaide SA Australia; ^6^ Department of Environmental Science The Connecticut Agricultural Experiment Station New Haven CT USA; ^7^ Australian Nuclear Science and Technology Organisation (ANSTO) Kirrawee DC NSW Australia; ^8^ Trace and Environmental DNA Lab School of Molecular and Life Sciences Curtin University Perth WA Australia

**Keywords:** Bayesian mixing models, food webs, groundwater, metagenomics, radiocarbon, stygofauna

## Abstract

Food web dynamics are vital in shaping the functional ecology of ecosystems. However, trophic ecology is still in its infancy in groundwater ecosystems due to the cryptic nature of these environments. To unravel trophic interactions between subterranean biota, we applied an interdisciplinary Bayesian mixing model design (multi‐factor BMM) based on the integration of faunal C and N bulk tissue stable isotope data (δ^13^C and δ^15^N) with radiocarbon data (Δ^14^C), and prior information from metagenomic analyses. We further compared outcomes from multi‐factor BMM with a conventional isotope double proxy mixing model (SIA BMM), triple proxy (δ^13^C, δ^15^N, and Δ^14^C, multi‐proxy BMM), and double proxy combined with DNA prior information (SIA + DNA BMM) designs. Three species of subterranean beetles (*Paroster macrosturtensis*, *Paroster mesosturtensis,* and *Paroster microsturtensis*) and their main prey items Chiltoniidae amphipods (AM1: *Scutachiltonia axfordi* and AM2: *Yilgarniella sturtensis*), cyclopoids and harpacticoids from a calcrete in Western Australia were targeted. Diet estimations from stable isotope only models (SIA BMM) indicated homogeneous patterns with modest preferences for amphipods as prey items. Multi‐proxy BMM suggested increased—and species‐specific—predatory pressures on amphipods coupled with high rates of scavenging/predation on sister species. SIA + DNA BMM showed marked preferences for amphipods AM1 and AM2, and reduced interspecific scavenging/predation on *Paroster* species. Multi‐factorial BMM revealed the most precise estimations (lower overall *SD* and very marginal beetles' interspecific interactions), indicating consistent preferences for amphipods AM1 in all the beetles' diets. Incorporation of genetic priors allowed crucial refining of the feeding preferences, while integration of more expensive radiocarbon data as a third proxy (when combined with genetic data) produced more precise outcomes but close dietary reconstruction to that from SIA + DNA BMM. Further multidisciplinary modeling from other groundwater environments will help elucidate the potential behind these designs and bring light to the feeding ecology of one the most vital ecosystems worldwide.

## Graphical abstract

Understanding of cryptic ecosystems is enabled by multi‐factor models and interdisciplinary research.

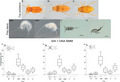

## 1 INTRODUCTION

Trophic dynamics provide vital information about ecological functioning (Lindeman, 1942; Polis & Winemiller, 2013; Start, 2018). Food web interactions shape ecological niche occupations and frame community dynamics (de Ruiter, Wolters, Moore, & Winemiller, 2005). The functionality of the trophic web is ultimately defined by intra‐ and interspecific interactions which shape biochemical patterns and energy flows within the ecosystems (Begon, Townsend, & Harper, 2006).

Both qualitative (Paine, 1980) and quantitative (Banasek‐Richter, Cattin, & Bersier, 2004) approaches have been applied in several ecosystems, the latter being more accurate but more challenging than the former (Kadoya, Osada, & Takimoto, 2012). Over the last four decades, isotope mixing models, such as IsoSource (Phillips & Gregg, 2001) or Bayesian mixing models (BMM, Parnell et al., 2013), have been increasingly used for quantitative reconstruction of dietary preferences. Both techniques aim to quantify unknown mixing contributions *via* measurement of the isotopic signals in consumers and food sources (Post, 2002).

Dietary proxies based on bulk carbon (δ^13^C) and nitrogen (δ^15^N) stable isotope analysis (SIA) are a powerful tool for studying trophic preferences and food web interactions (Fry, 2006 and references therein). Concurrently, radiocarbon (^14^C) forms a key tracer in untangling carbon incorporation and trophic pathways (Larsen, Yokoyama, & Fernandes, 2018). Metagenomics data, integrated with consumer and source abundances, provide semi‐quantitative *prior* information on dietary preferences that can refine statistical modeling (Chiaradia, Forero, McInnes, & Ramírez, 2014). BMM (i.e., MixSiar (Stock & Semmens, 2016)) allows integration of data from different disciplines such as biochemistry, genetics, and ecology, but the majority of trophic studies focus on stable isotopic frameworks. BMM FRUITS (Food Reconstruction Using Isotopic Transferred Signals, Fernandes, Millard, Brabec, Nadeau, & Grootes, 2014) enables a compelling multi‐factorial (multi‐proxy and multi‐prior) analysis for diet reconstruction. To date, FRUITS has been mainly employed in archaeological studies (e.g., Hamilton & Sayle, 2019) but rarely in freshwater ecology (e.g., Larsen et al., 2013).

Groundwaters are challenging systems for trophic ecology studies due to their poor accessibility and the largely unknown biochemical dynamics of subterranean organisms (Griebler, Malard, & Lefébure, 2014; Saccò, Blyth, Bateman, et al., 2019 and references therein). Stygofauna—aquatic obligate subterranean invertebrates—display low degrees of specialization driven by the lack of resources in groundwaters (e.g., Culver, 1994; Gibert & Deharveng, 2002; Hancock, Boulton, & Humphreys, 2005). However, investigations based on novel methodological approaches have recently started challenging this classic paradigm (e.g., Francois et al., 2020; Hutchins, Engel, Nowlin, & Schwartz, 2016), stressing the need for a refinement of feeding ecology studies in this context.

Sturt Meadows (SM) calcrete in Western Australia and its stygofaunal community provide a unique opportunity to compare isotopic trophic ecology models. SM stygofauna have been studied during the last 15 years *via* genetic (e.g., Cooper et al., 2007; Leys, Watts, Cooper, & Humphreys, 2003), ecological (e.g., Allford, Cooper, Humphreys, & Austin, 2008; Hyde, Cooper, Humphreys, Austin, & Munguia, 2018; Saccò et al., 2020), and isotopic (e.g., Bradford, Humphreys, Austin, & Cooper, 2014; Saccò, Blyth, Humphreys, et al., 2019) techniques, allowing a comprehensive understanding of the food web dynamics.

Three species of blind dytiscid beetles (*Paroster macrosturtensis*, *Paroster mesosturtensis,* and *Paroster microsturtensis*, all Watts & Humphreys 2006) lay at the top of the feeding chain, with amphipods *Scutachiltonia axfordi* (King, 2012) and *Yilgarniella sturtensis,* (King, 2012), and cyclopoids (Burmeister, 1834) and harpacticoids (G. O. Sars 1903) as their prey items. Feeding experiments and molecular analyses indicated that the beetles have marked preferences for the amphipod species *S. axfordi* followed by species‐specific predatory pressures on *Y. sturtensis* and copepods (Bradford, 2010; Bradford et al., 2014). Isotopic analysis (δ^13^C and δ^15^N SIA) of the three diving beetles revealed that the predatory pressures on both amphipods and copepods were also coupled with marginal interspecific predatory pressures on *Paroster* species (Saccò, Blyth, Humphreys, et al., 2019). However, the analysis of trophic interactions through such conventional approaches faces major challenges (Boecklen, Yarnes, Cook, & James, 2011), stressing the need for cost‐efficient model designs that allow the combination of data from multiple disciplines (Saccò, Blyth, Bateman, et al., 2019).

Here, we test whether the multi‐factor design of FRUITS models enables refinement of dietary preferences in the three species of subterranean aquatic beetles along with their food sources. The work aims to (a) evaluate the use of multi‐discipline and/or isotope only models in subterranean ecosystems and (b) provide recommendations on the use of isotopic techniques in groundwater ecology.

## 2 MATERIALS AND METHODS

### 2.1 Fieldwork, sample preparation, and faunal trophic ecology

Sampling occurred at a calcrete aquifer on Sturt Meadows pastoral station, in the Yilgarn, Western Australia. Stygofauna were sampled by haul nets (100 µm mesh size) from boreholes during two sampling campaigns carried out in July and November 2017. For further details about the sampling design, see Saccò et al. (2020). Specimens were sorted under a stereomicroscope to species level with reference to specific taxonomic keys (King, Bradford, Austin, Humphreys, & Cooper, 2012; Watts & Humphreys, 2009). All individuals from a single taxon were combined into one pool and washed with MilliQ water to remove external contaminants. Samples were oven dried at 60°C overnight, crushed to a fine powder, and stored at –20°C until analysis.

**FIGURE 1:**
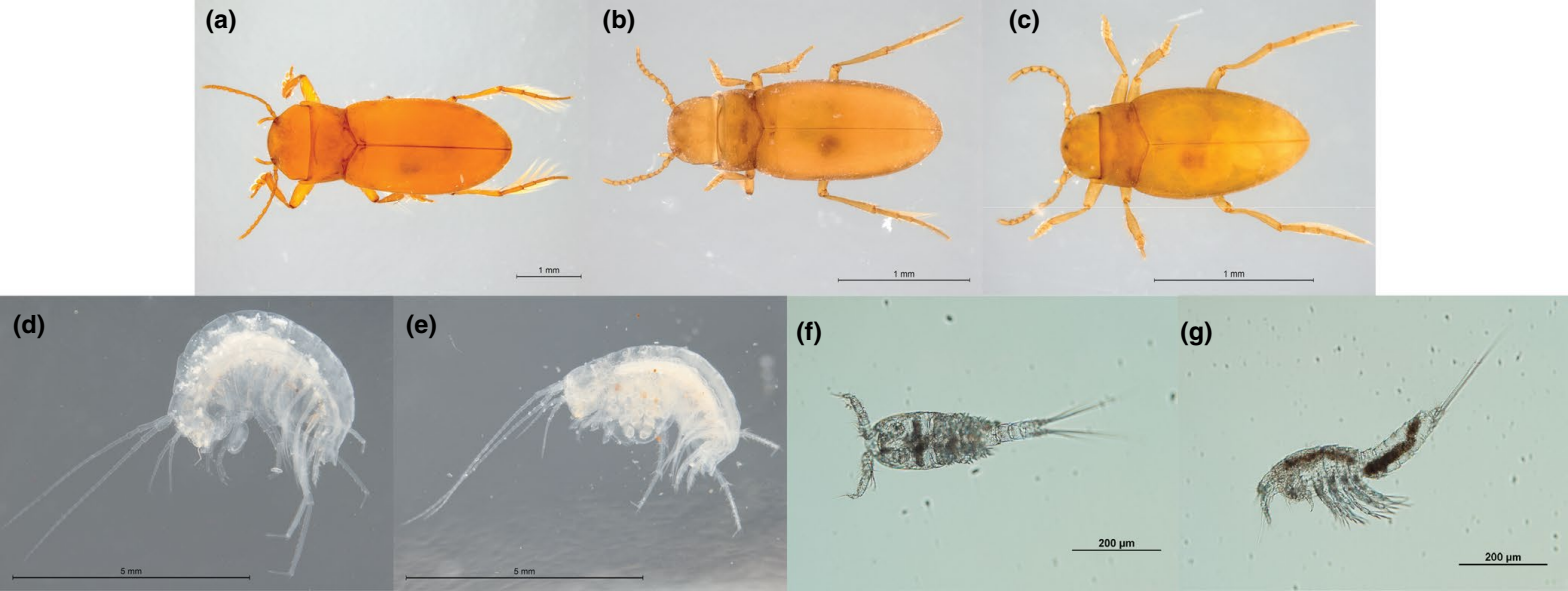
Photographs illustrating specimens belonging to the species (B) *Paroster macrosturtensis* (a), (M) *Paroster mesosturtensis* (b), (S) *Paroster microsturtensis* (c), (AM1) *Scutachiltonia axfordi* (d), and (AM2) *Yilgarniella sturtensis* (e)

Seven stygofaunal species were considered for the present study: three species of blind dytiscid diving beetles representing the top predators in the system (*P. macrosturtensis* (B‐big)*, P. mesosturtensis* (M‐medium), and *P. microsturtensis* (S‐small)) and four taxa of prey items: two species of amphipods (*S. axfordi* (AM1) and *Y. sturtensis* (AM2)) and two copepods (order Cyclopoida (C) and Harpacticoida (H)) (Figure 1). More details about the taxa morphology are provided in the Note S1.

Investigations on trophic habits based on genetic information (metabarcoding analysis) from previous studies at SM calcrete suggested that all three *Paroster* species feed on amphipod AM1 (ranging from 68% (B) to 28% (S) of their diet) more than the other groups and avoid intraspecific cannibalism. Specifically, while the diet of beetles B was dominated by amphipods (90% overall), beetles M and S preferred harpacticoids over amphipods AM2 (Bradford et al., 2014) (Table 1). Multi‐primer metabarcoding analyses (Ins16S and MZartCOI) on the three species indicated that occasional reciprocal scavenging/predation on sister species follows sister species‐specific patterns (Hyde, 2018) (Table S1).

**TABLE 1:** Trophic behaviors (predation and scavenging) based on prior metagenomics data on *Paroster macrosturtensis* (B), *Paroster mesosturtensis* (M), and *Paroster microsturtensis* (S)

	*P. macrosturtensis*	*P. mesosturtensis*	*P. microsturtensis*	References
Amphipods/copepods predation	AM1 > AM2 > H > C	AM1 > H > AM2 > C	AM1 > H > AM2 > C	[1],[2]
Beetles predation/Scavenging	*M* > S	S > B	*M* > B	[3], Table S1

Note

[1] Bradford (2010); [2] Bradford et al. (2014); [3] Hyde (2018).

Estimation of diet proportions of B, M, and S from isotopic analyses confirmed these trends, with amphipod AM1 being the preferred prey for all the three predator species (B: 25%; M: 27.4%; S: 25.4%) and copepods accounting for the 30% beetles' diets (Saccò, Blyth, Humphreys, et al., 2019).

### 2.2 Biochemical analysis

#### 2.2.1 Bulk SIA

C and N bulk stable isotopic analyses on homogenized stygofaunal samples (1.28 mg, 0.08–0.14 mg, and 0.63–2.79 mg per samples, respectively, see Saccò, Blyth, Humphreys, et al. (2019) for further details) were performed at the Australian Nuclear Science and Technology Organisation (ANSTO). Samples were loaded into tin capsules and analyzed by a continuous flow isotope ratio mass spectrometer (CF‐IRMS), model Delta V Plus (Thermo Scientific Corporation), interfaced with an elemental analyser (Thermo Fisher Flash 2000 HT EA, Thermo Electron Corporation) following Mazumder, Saintilan, Wen, Kobayashi, & Rogers, (2017). Carbon and nitrogen isotopic values are reported in per mil (‰) according to the standard delta (δ) notation, relative to the Vienna Peedee Belemnite (VPDB) and to atmospheric nitrogen (AIR), respectively. Results have an analytical precision of ±0.30‰. Results on the % of C and %N from bulk tissue were also obtained through the elemental analyser.

#### 2.2.2 Radiocarbon

Stygofaunal samples (~1 mg per sample for beetles (B, M and S) and amphipods (AM1 and AM2) and ~0.5 mg for copepods) were treated with dilute HCl (1 M) for 2 hr to remove carbonate contamination. Due to sample size constraints, cyclopoids and harpacticoids were combined in one sample, and therefore, a unique radiocarbon value (the first ever recorded for groundwater copepods) for both groups was obtained. The pre‐treated samples were combusted to CO_2_ and converted to graphite following Hua et al. (2001). ^14^C content of the sample graphite was determined using the accelerator mass spectrometry (AMS) STAR Facility at ANSTO (Sydney, Australia; Fink et al., 2004). Radiocarbon results are reported in Δ^14^C value in per mil (‰) relative to the absolute radiocarbon standard activity, and age was also assessed (with present being 1950 AD) (Stuiver & Polach, 1977),

### 2.3 Statistical analysis

**FIGURE 2:**
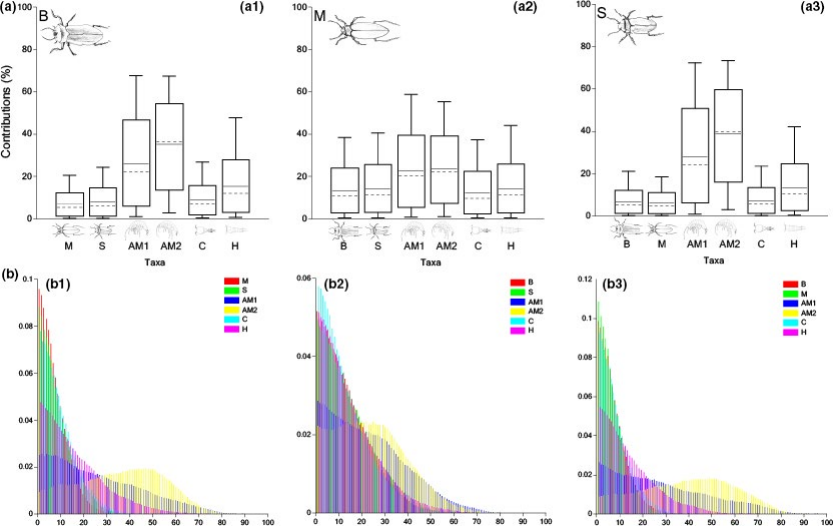
Estimated dietary contributions and probability distributions of B (a.1 and b.1), M (a.2 and b.2), and S (a.3 and b.3) for using bulk tissue δ^13^C and δ^15^N (SIA BMM). Boxes and whiskers indicate 68% and 95% credible intervals, respectively. Horizontal continuous lines indicate the estimated mean, and dashed lines refer to the median. Refer to Table S2 for the specific contribution mean values

Relative contributions from dietary items were estimated using the software FRUITS version 2.1.1 beta (Fernandes et al., 2014). FRUITS allows quantification of the dietary proportions of food sources (defined as “sources”) among consumers (defined as “targets”) *via* isotopic quantitative signals (defined as “proxies”). The model incorporates variance associated with isotopic measurements from sources and targets, trophic discrimination offsets, and allows incorporation of prior information to refine the analysis (defined as “priors”). FRUITS models generate a BUGS (Bayesian inference Using Gibbs Sampling) coding that is then transferred to OpenBUGS package, a software commonly used for Bayesian probability modeling (Lunn, Thomas, Best, & Spiegelhalter, 2000). Markov chain Monte Carlo (MCMC) simulations allow generation of posterior distributions associated with credible intervals (Gilks, Richardson, & Spiegelhalter, 1996). BMM was applied to test the potential changes in dietary preferences. Figure 2 depicts the statistical design used for the present study.

The multi‐factor BMM was run using δ^13^C and δ^15^N values from bulk tissue analysis. Specific trophic discrimination factors are not yet available for stygofauna, so in all bulk tissues we used the widely accepted discrimination values of 3.46 ± 2‰ for nitrogen and 0.5 ± 1‰ for carbon (Zanden & Rasmussen, 2001). The third proxy was radiocarbon data (Δ^14^C). Coupled with stable isotope proxies, this has the potential to provide more discriminatory carbon fingerprints between sources and consumers (Larsen et al., 2018). Δ^14^C is free from isotopic fractionation due to the internal correction by the δ^13^C of −25‰ (Ishikawa, Hyodo, & Tayasu, 2013). This “triple proxy approach” (δ^13^C, δ^15^N, and Δ^14^C) was coupled with inputs from prior information (both from sources and targets) from metagenomics analyses to create a multi‐factorial design (hereafter defined as “multi‐factor BMM”). Despite the differences in research targets between isotope ecology (focused on “who assimilates whom”) and metagenomics (focused on “who eats whom”) (Ishikawa, 2018), the combination of these data within the framework of BMM has been proved to allow crucial refining of diet analyses in a vast number of studies (e.g., Franco‐Trecu et al., 2013; Galvan, Sweeting, & Polunin, 2012; Matley et al., 2018; Traugott, Kamenova, Ruess, Seeber, & Plantegenest, 2013). Dietary contributions estimated *via* multi‐factor BMM were compared with a classic δ^13^C with δ^15^N SIA BMM, conventional SIA coupled with genetic prior information (SIA + DNA BMM) and the “triple proxy approach” alone (multi‐proxy BMM).

## 3 RESULTS

### 3.1 Stable isotope and radiocarbon characterization

Among beetles, % C from bulk tissue was directly proportional to their body size, and amphipods showed values comparable to beetles B and M. Contrarily, copepods (C and H) indicated very low percentages of carbon coupled with the highest % of N out of the seven groups (Table 2). δ^13^C values of beetles and amphipods were close each other, ranging from −24.55 ± 0.3‰ (AM2) to −23 ± 0.19‰ (B), while copepods (both C and H) showed lower values. Contrarily, δ^15^N values of beetles were the highest among the potential prey items, ranging from 15.43 ± 0.53‰ (M) to 14.4 ± 0.3, while amphipods displayed lower values (Table 2). As already commented in Saccò, Blyth, Humphreys, et al. (2019), copepods had high δ^15^N, suggesting alternative metabolic pathways compared to the amphipods. Average Δ^14^C values ranged from −5.6 ± 5.6‰ (C/H) to 37 ± 2.2‰ (M) (Table 2). All the radiocarbon samples indicated modern carbon sources.

**TABLE 2:** Carbon and nitrogen isotopic ratios for δ^13^C and δ^15^N SIA (together with the % of C and N) and Δ^14^C values (in ‰)

Taxa	ID	%C	δ^13^C	%N	δ^15^N	Δ^14^C
*Paroster macrosturtensis*	B	60.07	−23 ± 0.19	7.20	14.66 ± 0.27	32.90 ± 2.30
*Paroster mesosturtensis*	M	56.65	−23.37 ± 0.19	7.09	15.43 ± 0.53	37 ± 2.20
*Paroster microsturtensis*	S	39.80	−23.60 ± 0.30^1^	5.30	14.40 ± 0.30^1^	22.90 ± 3.10
*Scutachiltonia axfordi*	AM1	57.70	−24.14 ± 0.30^1^	2.80	10.71 ± 0.30^1^	19.90 ± 4.80
*Yilgarniella sturtensis*	AM2	58.30	−24.55 ± 0.30^1^	2.80	9.99 ± 0.30^1^	−3.70 ± 3.60
Cyclopoida	C	0.11	−20.45 ± 0.30^1^	13.90	13.90 ± 0.30^1^	−5.60 ± 5.60^2^
Harpacticoida	H	0.10	−20.60 ± 0.30^1^	11.90	11.90 ± 0.80	−5.60 ± 5.60^2^

Note

Stable isotopic data previously reported in Saccò, Blyth, Humphreys, et al. (2019).

^1^ Accuracy of the CF‐iRMS (unique runs).

^2^ Homogenized values.

### 3.2 Dietary contributions

#### 3.2.1 SIA BMM

Diet contributions of beetles B were dominated by amphipods AM2 (35.21 ± 18.19%) and AM1 (25.89 ± 18.86%), followed by harpacticoids (H: 15.41 ± 12.73%) and cyclopoids (C: 8.80 ± 7.27%) (Figure 2a.1). Beetles M showed higher scavenging/predation on sister species S (14.15 ± 11.17%) and B (13.32 ± 10.45%) and lower proportions of amphipods (equally distributed between AM1 and AM2, accounting for the ~46% of the total) when compared to beetles B (Figure 2a.2). Contrarily, scavenging/predation on sister species was marginal within diets of beetles S (always below 7%, Table S2), with amphipods AM2 (38.75 ± 19.66%) being the main prey item followed by amphipods AM1 (27.83 ± 20.43%) (Figure 2a.3).

#### 3.2.2 Multi‐proxy SIA BMM

**FIGURE 3:**
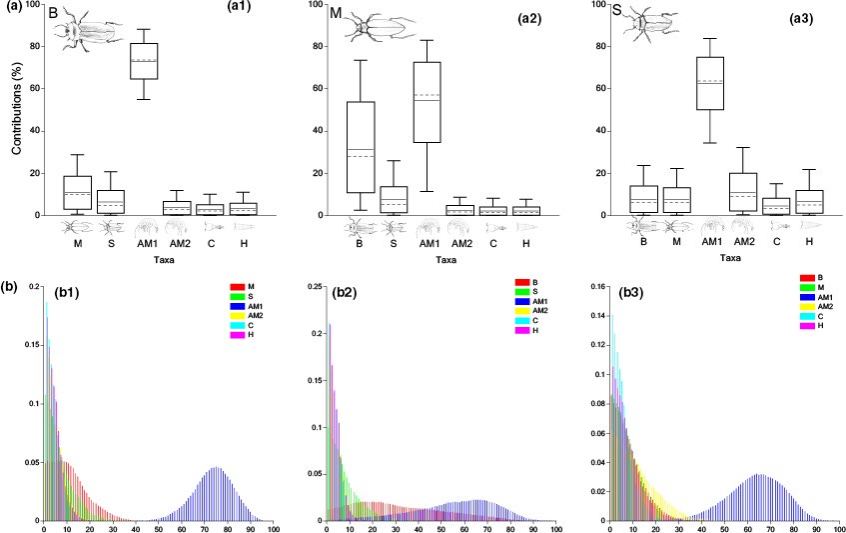
Estimated dietary contributions and probability distributions of B (a.1 and b.1), M (a.2 and b.2), and S (a.3 and b.3) for using bulk tissue δ^13^C and δ^15^N combined with Δ^14^C data (multi‐proxy BMM). Boxes and whiskers indicate 68% and 95% credible intervals, respectively. Horizontal continuous lines indicate the estimated mean, and dashed lines refer to the median. Refer to Table S2 for the specific contribution mean values

Diet estimations of beetles B were markedly dominated by amphipods AM1, which composed almost two thirds of the overall contributions (73.07 ± 8.44%). Sister species scavenging/predation was preferred over predation of amphipod AM2 (AM2 accounting for just 3.63 ± 3.26%, Table S2) and copepods (C and H accounting together for ~6%) (Figure 3a.1). Dietary estimations for beetles M indicated conspicuous scavenging/predation on sister species B (31.36 ± 19.78%) while amphipod AM2 and copepods played a very marginal role (Table S2, Figure 3a.2). The diet of beetles S was dominated by amphipods AM1 (62.60 ± 12.68%) and AM2 (11.07 ± 8.85%), with sister species scavenging/predation sitting at the same secondary level as copepods' predation (Figure 3a.3).

#### 3.2.3 SIA + DNA BMM

**FIGURE 4:**
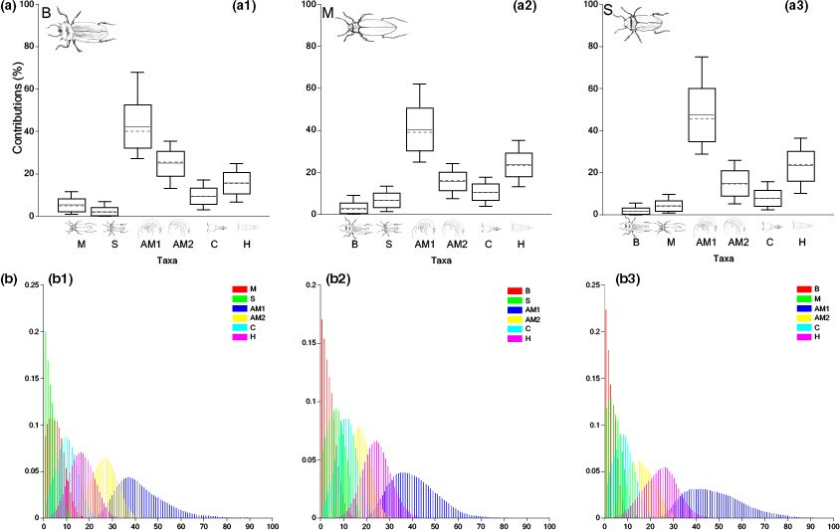
Estimated dietary contributions and probability distributions of B (a.1 and b.1), M (a.2 and b.2), and S (a.3 and b.3) for using bulk tissue δ^13^C and δ^15^N combined with prior metabarcoding data (SIA + DNA BMM). Boxes and whiskers indicate 68% and 95% credible intervals, respectively. Horizontal continuous lines indicate the estimated mean, and dashed lines refer to the median. Refer to Table S2 for the specific contribution mean values

Amphipods contributed the most to diet of beetles B (AM1 (42.15 ± 10.42%) and AM2 (25.01 ± 5.80%)), followed by copepods (H: 15.67 ± 4.80%; C: 9.53 ± 3.68%) and very marginal scavenging/predation of sister species (beetles M and S accounting for ~7.6% together, Table S2) (Figure 4a.1). Diets of M and S followed the same trends, characterized by a dominance of amphipods AM1 (accounting for 40.28 ± 9.84% and 47.45 ± 12.20% respectively), high proportions of harpacticoids (~23.6% for both beetle species, Table S2), few copepods (10.62 ± 3.66% (M) and 8.03 ± 3.59% (S)), and marginal Dytiscidae scavenging/predation (always below 9%) (Figure 4a.2,3).

#### 3.2.4 Multi‐factor SIA BMM

**FIGURE 5:**
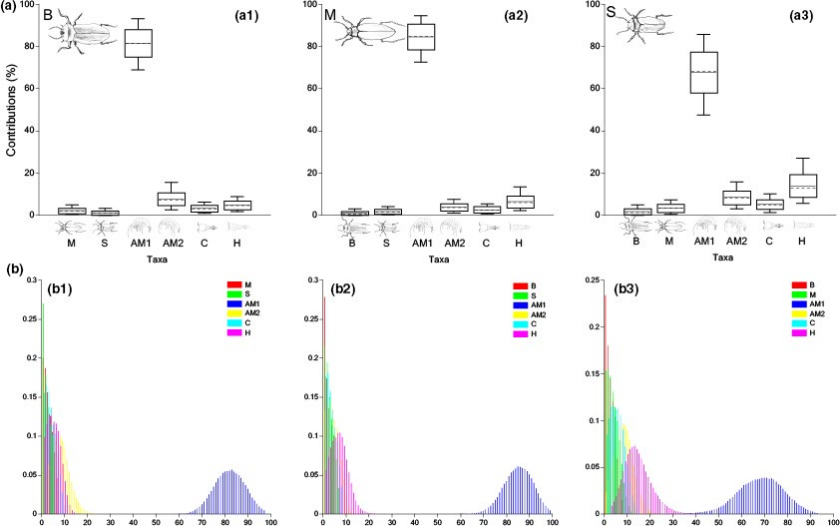
Estimated dietary contributions and probability distributions of B (a.1 and b.1), M (a.2 and b.2), and S (a.3 and b.3) for using bulk tissue δ^13^C and δ^15^N, combined with Δ^14^C and prior metabarcoding data (multi‐factor BMM). Boxes and whiskers indicate 68% and 95% credible intervals, respectively. Horizontal continuous lines indicate the estimated mean, and dashed lines refer to the median. Refer to Table S2 for the specific contribution mean values

Beetles' dietary makeup showed similar patterns among the three species. Amphipods AM1 were markedly the preferred prey items (B: 81.47 ± 6.23%; M: 84.49 ± 5.82%; S: 67.74 ± 9.65%), and species AM2 played a minor role, accounting for 7.64 ± 3.20% (for beetles B, being AM2 their second preferred prey item, same as for S) and 3.85 ± 1.67% (M) (Figure 5a.1,2,3). Similar to the results from SIA + DNA BMM, harpacticoids (H) were the second preferred prey items for beetles M (6.49 ± 2.89%) and S (13.87 ± 5.51%), and interspecific scavenging/predation was very marginal (Table S2).

## 4 DISCUSSION AND CONCLUSIONS

### 4.1 Multi‐factor mixing models in calcretes

Our findings indicate that the four designs of BMM employed result in different predictions for the diet preferences of stygofaunal beetle species. Standard stable isotope mixing models using bulk tissue (SIA BMM) showed a tendency toward homogeneously distributed proportions and high *Paroster* interspecies interactions (with proportions up to ~27% in beetles M). However, uncertainty of the modeled estimations was very high (*SD* values reaching values up to 20.43%, Table S2), indicating poor fitting.

Incorporation of radiocarbon proxy data (Δ^14^C) as a source tracer allowed tailoring of the trophic interactions around species‐specific shifts in carbon assimilations. Overall, the small negative Δ^14^C values for amphipod AM2 and copepods (C and H) suggested that the carbon involved in their biochemical cycles was formed before 1950, while the positive values detected for beetles revealed modern sources indicative of high positions in the trophic chain (Hyodo et al., 2008). To our best knowledge, radiocarbon data from groundwater copepods were included for the first time in this study. Due to sample size constraints, cyclopoids and harpacticoids were combined together in one lot and same Δ^14^C values were considered for both groups. Values of δ^13^C and % C of cyclopoids were comparable to those of harpacticoids (Table 2), indicating comparable carbon pathways. This aspect, combined with the small intra‐seasonal variability of Δ^14^C values reported for crustaceans (e.g., Keaveney, Reimer, & Foy, 2015), provides us with confidence on the representativeness of our dataset.

When compared to SIA BMM, multi‐proxy BMM illustrated reduced overall uncertainty of the estimations (apart from AM1 estimations for beetles M and S and scavenging/predation from M to beetles B, *SD* values were always below 10%, Table S2) and pinpointed AM1 as the vital prey item in *Paroster* diets. However, high rates of scavenging/predation on sister species, reaching up to 31.36% for diets of beetles M, were also found. As per SIA BMM, these results challenge our previous knowledge of the feeding ecology of the subterranean dytiscid beetles. Groundwaters provide very stable environments characterized by low resources, and evolutionary (Bradford et al., 2014) and ecological (Hyde, 2018) forces are expected to drive very marginal interspecies predation among top predators at SM calcrete. As a result, we argue here that the outcomes from both SIA and multi‐proxy BMM provide imperfect beetles' diet estimations at SM calcrete and advocate for the integration of further data for more reliable modeling.

Beetles' dietary makeup through SIA + DNA BMM was more precise than the outcomes from stable isotopes only design, with *SD* values always below 13% (Table S2). Moreover, narrower probability distributions (Figure 4b.1,2,3) compared with the results from SIA BMM (Figure 2b.1,2,3) indicated better fitting. Predation on amphipods and copepods was always preferred over sister species scavenging/predation (Figure 4a.1,2,3), an outcome in line with previous investigations at SM calcrete (Bradford, 2010; Bradford et al., 2014). Compared to previous designs, the multi‐factorial model (multi‐factor BMM) showed reduced levels of uncertainty (*SD* values always below 10%, Table S2) and suggested that subterranean beetles exert preferential predatory pressures on AM1 coupled with extremely marginal (always below 5% combining two groups) interspecific interactions.

Our outcomes from multi‐factor modeling, together with the results from SIA + DNA BMM, indicated that subterranean beetles at Sturt Meadows lack trophic niche partitioning, as already suggested by Bradford et al. (2014). While seemingly counterintuitive and in contrast to the classic subterranean ecology paradigm of opportunistic feeding traits, our results coincide with the conclusions drawn by Francois et al. (2016). This indicated that widely reported low metabolic rates and resource‐gathering abilities might play a role in releasing the constraints on trophic specialization underground. Resulting from these eco‐evolutionary dynamics, groundwater fauna is suggested to display feeding habits focused on more ubiquitous resources (i.e., sedimentary biofilm, prey items, etc.) rather than being driven by selective forces toward generalist strategies. At SM calcrete, species‐specific ethological (i.e., group feeding) and physiological features (high efficiency in metabolic activation processes in the smaller species such as M and S, see Jones, Cooper, & Seymour, 2019) might have played a role in homogenizing the trophic habits of the three top predator species studied. However, additional studies will be necessary to elucidate the specific aspects of these cryptic behaviors and their linkage to evolutionary dynamics.

Overall, multi‐factor designs, followed by SIA + DNA BMM, offered the highest level of precision for the interpretation of species‐specific foraging ecology patterns among calcrete subterranean beetles. However, further work is needed to determine which model is most accurate. Indeed, prior information in the posterior distribution is likely to have played a key role in shaping our patterns, confirming that estimation of diet benefits from genetic data on potential prey (Chiaradia et al., 2014). Given that the majority of information about groundwater fauna comes from genetic investigations (i.e., metabarcoding), BMM allows novel coupling between isotopic (quantitative) and molecular (qualitative or semi‐quantitative) data and has the potential to bring light to the mechanisms sustaining biodiversity in of one of the largest and most understudied ecosystems in the world. This combination of methodologies remains under‐exploited (Majdi et al., 2018), with the present study still limited to one arid zone calcrete system. Investigations involving different groundwater environments (i.e. alluvial aquifers, karst, etc.) will help elucidate the potential behind the integration of data from different disciplines into isotopic ecology studies on subterranean fauna.

### 4.2 Defining the (realistic) current isotopic design in groundwater studies

The study of stygofaunal foraging ecology through isotopic techniques is gaining prominence as an analytical approach to dig into groundwater energy flows and trophic niche interactions (Saccò, Blyth, Bateman, et al., 2019). However, the current technical and analytical advances seen in the broader field of isotopic ecology are frequently coupled with increased price. As a result, a balance between cost and precision of outcome must be achieved.

To date, the vast majority of groundwater isotopic food web studies involve conventional bulk tissue SIA (e.g., Hartland, Fenwick, & Bury, 2011; Simon, Benfield, & Macko, 2003). However, δ^13^C and δ^15^N measurement alone, despite being the cheapest analytical approach available, have been reported to be only partially accurate due to the mixing of biochemical fractionation pathways (Newsome, Fogel, Kelly, & del Rio, 2011 and references therein). Our results concur with this observation, indicating that isotopic trophic studies in groundwaters using classic SIA designs are potentially exposed to misinterpretation (Table 3).

**TABLE 3:** Cost–benefit evaluation of the four techniques employed in this study

Analytical design	Cost	Precision	References	Recommendation
SIA	$	#	[1],[2]	*
SIA + DNA	$$	###	[3],[4]	****
SIA + ^14^C	$$$	##	[5],[6]	**
SIA + ^14^C + DNA	$$$$	####	[7]	***

Note

[1] Zanden and Rasmussen (2001); [2] Bowes and Thorp (2015); [3] Elbrecht, Vamos, Meissner, Aroviita, and Leese (2017); [4] Gardham et al. (2014); [5] Chapple (2010); [6] Pilcher (2003); [7] Larsen et al. (2018).

Cost: $ low, $$ moderate, $$$ high, and $$$$ very high.

Precision: # low, ## moderate, ### high, and #### very high.

Recommendation: * low, **moderate, ***high, and **** very high.

The incorporation of a third proxy (Δ^14^C in our study) into BMM is unexplored in groundwater feeding ecology studies, probably due to budgetary constraints (^14^C analysis has a cost that can exceed ten times SIA) and analytical issues (low carbon inputs/content provide an additional challenge in subterranean ecosystems). While the patterns generated through our triple‐proxy design showed reduced variability of the diet estimations (and allowed improved tracing of the carbon flow), they did not align with the marginal role of beetles' interspecific interactions indicated by the previous studies carried out at SM calcrete (Bradford, 2010; Bradford et al., 2014; Hyde, 2018). Overall, this scenario suggests that the only partial increase in precision does not merit the additional cost of the triple‐proxy design without additional prior information (Table 3).

Stygofauna can display cryptic feeding habits (Stoch, 1995) which are hard to investigate in mesocosm experiments, and genetically based biological characterizations can be crucial in identifying diet preferences under natural conditions (Saccò, Blyth, Bateman, et al., 2019). Once incorporated into BMM, this combination of data from independent sources enables comparison of datasets and can constrain the systemic biases of each separate technique (Chiaradia et al., 2014). Overall, we suggest that reconciliation of the trade‐off between the cost (price) and benefit (precision) in groundwater food web studies can be achieved by incorporating metabarcoding data into a model with conventional SIA. The most precise outcomes are obtained by integration of triple isotope proxy and DNA data, but overall dietary reconstruction was close to that from bulk tissue isotopes coupled with genetic prior information. Therefore, SIA + DNA is recommended generally, with full multi‐factorial approaches used where operational costs are not a significant constraint.

Further advances, including specific investigations on the variability of the trophic discrimination factors (McMahon & McCarthy, 2016) for stygofauna, will enhance the biochemical understanding of trophic pathways and help refine analyses (Parnell, Inger, Bearhop, & Jackson, 2010). Recent more expensive novel analytical approaches such as compound specific isotopic analyses offer to refine foraging ecology studies and overcome some of the homogenization issues in bulk tissue SIA (Chikaraishi et al., 2009; Larsen et al., 2013; Steffan et al., 2013). The combination of SIA and CSIA has recently gained prominence in the broad literature (Potapov, Tiunov, Scheu, Larsen, & Pollierer, 2019) and has been applied in the field of groundwater ecology (Saccò, Blyth, Humphreys, et al., 2019). However, while these techniques are a cornerstone in trophic studies, conventional SIA approaches are likely to be widely used in the near future due to constraints of budget and technical limitations in CSIA. Despite its averaging of biochemical fractionation pathways, bulk tissue SIA, when integrated with prior qualitative information on the feeding habits, still allows elucidation of the food web interactions. When applied to groundwater ecology studies, we believe that these designs have the potential to enable affordable and reasonably accurate interpretation of the stygofaunal foraging ecology.

## CONFLICT OF INTEREST

None declared.

## AUTHOR CONTRIBUTIONS

**Mattia Saccò:** Conceptualization (lead); data curation (lead); formal analysis (lead); investigation (lead); methodology (lead); writing – original draft (lead). **Alison J. Blyth:** Funding acquisition (lead); project administration (equal); resources (equal); supervision (lead); validation (equal); writing – review & editing (equal). **William F. Humphreys:** Funding acquisition (lead); investigation (equal); supervision (equal); writing – review & editing (equal). **Steven J. B. Cooper:** Funding acquisition (lead); project administration (equal); resources (lead); writing – review & editing (equal). **Andrew D. Austin:** Funding acquisition (lead); project administration (lead); resources (equal); writing – review & editing (equal). **Josephine Hyde:** Writing – review & editing (equal). **Debashish Mazumder:** Data curation (equal); formal analysis (equal); resources (equal); writing – review & editing (equal). **Quan Hua:** Data curation (equal); formal analysis (equal); resources (equal); supervision (equal); writing – review & editing (equal). **Nicole E. White:** Data curation (equal); formal analysis (equal); methodology (equal); resources (equal); writing – review & editing (equal). **Kliti Grice:** Funding acquisition (equal); resources (equal); supervision (equal); writing – review & editing (equal).

## Supporting information

Supplementary Material

## Data Availability

All additional data are available in the Supporting Information and will be archived in the Dryad repository (https://doi.org/10.5061/dryad.2z34tmpj7).
