# Supplementary material for: Refining trophic dynamics through multi‐factor Bayesian mixing models: A case study of subterranean beetles
Source: Ecol Evol. 2020 Jul 20;10(16):8815–26. doi: 10.1002/ece3.6580 (PMC7452819; doi:10.1002/ece3.6580)
Supplement: Supplementary file 1 — Supplementary Material [file ECE3-10-8815-s001.docx]

**SUPPORTING INFORMATION**

**Note S1**

B, M and S are sister species characterised by high genetic diversity (Guzik, Cooper, & Austin 2009), with analyses suggesting they diversified in groundwater from a stygobiont ancestor (Langille, Tierney, & Cooper, 2019). The species live permanently within the groundwater, where they respire directly through their cuticle (Jones et al, 2019). They usually show predatory and opportunistic (i.e. scavenger, see Hyde, 2018) feeding behaviours (Saccò et al., 2020) and are characterised by different corporal features. B has the largest body (3.6-4.1 mm) with thin antennae and prominent elytral shoulders (Fig. 1a). M is 2.0-2.3 mm, with a relatively small head and pronotum, and small posterior legs (Fig. 1b), the specimens of this species showing marked sexual dimorphism. S is 1.7-1.8 mm with boat-shaped bodies and mesofemurs (strongly sinuated) and metafemurs (heavily scalloped) with distinctive characters (Watts & Humphreys 2009) (Fig. 1c).

Amphipods AM1 and AM2 belong to the family Chiltoniidae that, together with *Scutachiltonia bradfordae* (King et al., 2012) (AM3), inhabit the Sturt Meadows calcrete. They have distinct stygobiontic traits: elongated antennae and uropods, and lack of both pigmentation and eyes. Molecular phylogenetic analyses (Bradford, Adams, & Cooper 2010) indicate that these amphipods are not sister clades, but belong to three different lineages genetically more similar to other Yilgarn and non-Yilgarn amphipods than to each other.

AM1 is the largest species and its distinctive traits are elongated coxae and setose pereiopods (Fig. 1d). It is a unique morphotype in calcrete aquifers and is endemic to the Sturt Meadows calcrete (King et al., 2012). AM2 has the least stygobiotic-like morphology with medium size body features (Fig. 1e). AM3 is the rarest species in the calcrete (King et al. 2012), and it was not included in the isotopic analysis due to insufficient material.

The orders Cyclopoida (C) and Harpacticoida (H) belong to the two taxa of copepods that most successfully invaded inland groundwater worldwide (Galassi, 2001) and display peculiar traits adaptive to the interstitial life. Cyclopoids differ from other copepods by having first antennae shorter than the length of the head and thorax, and uniramous second antennae (Lowry, 1999) (Fig. 1f). Harpacticoids display a very short pair of first antennae, biramous second pair of antennae and a wide abdomen, with a worm-like body (Barnes, 1987) (Fig. 1g).

**Additional references**

Barnes, R. D. (1987). Invertebrate zoology (No. Ed. 5). WB Saunders company.

Bradford, T., Adams, M., Humphreys, W. F., Austin, A. D., & Cooper, S. J. B. (2010). DNA barcoding of stygofauna uncovers cryptic amphipod diversity in a calcrete aquifer in Western Australia’s arid zone. Molecular Ecology Resources, 10(1), 41-50.

Galassi, D. M. (2001). Groundwater copepods: diversity patterns over ecological and evolutionary scales. Hydrobiologia, 453(1), 227-253.

Guzik, M. T., Cooper, S. J. B., Humphreys, W. F., & Austin, A. D. (2009). Fine‐scale comparative phylogeography of a sympatric sister species triplet of subterranean diving beetles from a single calcrete aquifer in Western Australia. Molecular Ecology, 18(17), 3683-3698.

Langille, B. L., Tierney, S. M., Austin, A. D., Humphreys, W. F., & Cooper, S. J. (2019). How blind are they? Phototactic responses in stygobiont diving beetles (Coleoptera: Dytiscidae) from calcrete aquifers of Western Australia. Austral Entomology, 58(2), 425-431.

Lowry, J. K. (1999). Crustacea, the Higher Taxa: Description, Identification, and Information Retrieval. Version 2 October 1999.

**TABLE S1** Results from multi-primer (Ins16S and MZartCOI) metabarcoding analyses on B, M and S. The methodology followed for the obtention of the results is described below.

Extraction of DNA: a total of 15 individuals for each of the three *Paroster* species (*P. macrosturtensis* (B)*, P. mesosturtensis* (M)*, P. microsturtensis* (S)) were sorted into triplicate stygobitic pools (i.e. 5 individuals per pool) for DNA extraction. Prior to DNA extraction, stygobitic animals (5 individuals per pool; *n*=18) were placed in a petri dish containing ultrapure water and UV sterilised for 10 minutes to eliminate any potential eDNA that may be contained on the exoskeleton. Immediately post-UV treatment, the animals were placed into tissue lysis tubes with 180 µL ATL and 20 µL Proteinase K and homogenised using Minilys® tissue homogeniser (ThermoFisher Scientific, Australia) at high speed for 30 seconds. Lysis tubes, inclusive of one laboratory control, were incubated at 56°C using an agitating heat block (Eppendorf ThermoStat™ C, VWR, Australia) for 5 hours. Following incubation, DNA extraction was carried out using DNeasy Blood and Tissue Kit (Qiagen; Venlo, Netherlands eluted off the silica column in 50 µL AE buffer.

PCR: The quality and quantity of DNA extracted from each stygobitic pool was measured using quantitative PCR (qPCR), insect 16S and COI genes. PCR reactions to assess the quality and quantity of the DNA target of interest were carried out *via* qPCR (Applied Biosystems [ABI], USA) in 25 µL reaction volumes consisting of 2 mM MgCl_2_ (Fisher Biotec, Australia), 1 x PCR Gold Buffer (Fisher Biotec, Australia), 0.4 µM dNTPs (Astral Scientific, Australia), 0.1 mg bovine serum albumin (Fisher Biotec, Australia), 0.4 µM of each primer (MZArtF and MZArtR Zeale, Butlin, & Jones 2011; Ins16S_1shortF and Ins16S_1shortR Clarke, Soubrier, & Cooper 2014), 0.2 µL of AmpliTaq Gold (AmpliTaq Gold, ABI, USA), and 2 µL of template DNA (Neat, 1/10, 1/100 dilutions). The cycling conditions were: initial denaturation at 95°C for 5 minutes, followed by 40 cycles of 95°C for 30 seconds, 52°C for 30 seconds, 72°C for 30 seconds, and a final extension at 72°C for 10 minutes.

DNA extracts that successfully yielded DNA of sufficient quality, free of inhibition, as determined by the initial qPCR screen (detailed above), were assigned a unique 6-8bp multiplex identifier tag (MID-tag) with the 16S and COI primer set. Independent MID-tag qPCR for each stygobitic pool were carried out in 25 µL reactions containing 1 x PCR Gold Buffer, 2.5 mM MgCl_2_, 0.4 mg/mL BSA, 0.25 mM of each dNTP, 0.4 µM of each primer, 0.2 µL AmpliTaq Gold and 2-4 µL of DNA as determined by the initial qPCR screen. The cycling conditions for qPCR using the MID-tag primer sets were as described above. MID-tag PCR amplicons were generated in duplicate and the library was pooled in equimolar ratio post-PCR for DNA sequencing. The final library was size selected (160-600bp) using Pippin Prep (Sage Sciences, USA) to remove any MID-tag primer-dimer products that may have formed during amplification. The final library concentration was determined using a QuBitTM 4 Fluorometer (Thermofischer, Australia) and sequenced using a 300 cycle V2 kit on an Illumina MiSeq platform (Illumina, USA).

MID-tag sequence reads obtained from the MiSeq were sorted (filtered) back to the stygobitic pool based on the MID-tags assigned to each DNA extract using Geneious v10.2.5. MID-tag and primer sequences were trimmed from the sequence reads allowing for no mismatch in length or base composition. Each stygobitic pool for the two genes amplified were searched using BLASTn (Altschul, Gish, & Lipman 1990), against the NCBI GenBank nucleotide database to enable taxonomic identification. This was automated in the internet-based bioinformatics workflow environment through Pawsey Supercomputing (REF). The BLAST results obtained were imported into MEtaGenome Analyzer v4 (MEGAN; Huson, Auch, & Schuster 2007), where they were taxonomically assigned using the LCA-assignment algorithm (min. bit score = 65.0, top percentage = 5%, min. support = 5).

**Additional references**

Altschul, S. F., Gish, W., Miller, W., Myers, E. W., & Lipman, D. J. (1990). Basic local alignment search tool. Journal of molecular biology, 215(3), 403-410.

Clarke, L. J., Soubrier, J., Weyrich, L. S., & Cooper, A. (2014). Environmental metabarcodes for insects: in silico PCR reveals potential for taxonomic bias. Molecular ecology resources, 14(6), 1160-1170.

Huson, D. H., Auch, A. F., Qi, J., & Schuster, S. C. (2007). MEGAN analysis of metagenomic data. Genome research, 17(3), 377-386.

Zeale, M. R., Butlin, R. K., Barker, G. L., Lees, D. C., & Jones, G. (2011). Taxon‐specific PCR for DNA barcoding arthropod prey in bat faeces. Molecular ecology resources, 11(2), 236-244.

**TABLE S2** Beetles’ (B, M and S) dietary contributions values (in %).
